# Tumor-based gene expression biomarkers to predict survival following curative intent resection for stage I lung adenocarcinoma

**DOI:** 10.1371/journal.pone.0207513

**Published:** 2018-11-20

**Authors:** Alisson Clemenceau, Nathalie Gaudreault, Cyndi Henry, Paula A. Ugalde, Catherine Labbé, Michel Laviolette, Philippe Joubert, Yohan Bossé

**Affiliations:** 1 Institut universitaire de cardiologie et de pneumologie de Québec, Quebec, Canada; 2 Department of Molecular Biology, Medical Biochemistry and Pathology, Laval University, Quebec, Canada; 3 Department of Molecular Medicine, Laval University, Quebec, Canada; Universitat de Barcelona, SPAIN

## Abstract

**Background:**

Prognostic biomarkers are needed in clinical setting to predict outcome after resection for early-stage lung adenocarcinoma. The goal of this study is to validate tumor-based single-gene expression biomarkers with demonstrated prognostic value in order to move them along the clinical translation pipeline.

**Methods:**

Prognostic genes were selected from the literature and the best candidates measured by quantitative real-time polymerase chain reaction (qPCR) in tumors of 233 patients with stage I adenocarcinoma. Significant prognostic genes were then validated in an independent set of 210 patients matching the first set in terms of histology, stage, and clinical data.

**Results:**

Eleven genes with demonstrated prognostic value were selected from the literature. Complementary analyses in public databases and our own microarray dataset led to the investigation of six genes associated with good (*BTG2*, *SELENBP1* and *NFIB*) or poor outcome (*RRM1*, *EZH2* and *FOXM1*). In the first set of patients, *EZH2* and *RRM1* were significantly associated with better survival on top of age, sex and pathological stage (*EZH2* p = 3.2e-02, *RRM1* p = 5.9e-04). The prognostic values of *EZH2* and *RRM1* were not replicated in the second set of patients. A trend was observed for both genes in the joint analyses (n = 443) with higher expression associated with worse outcome.

**Conclusion:**

Adenocarcinoma-specific mRNA expression levels of *EZH2* and *RRM1* are associated with poor post-surgical survival in the first set of patients, but not replicated in a clinically and pathologically matched independent validation set. This study highlights challenges associated with clinical translation of prognostic biomarkers.

## Introduction

Surgery remains the first line of treatment for operable and resectable stage I pulmonary adenocarcinoma. This histological subtype and stage represents the largest proportion of patients undergoing surgical intervention. For these patients, the standard postoperative approach is observation [1]. However, disease recurrence is still a persistent problem for this population [2]. There is thus an urgent need to identify postoperative stage I adenocarcinoma lung cancer patients at high risk of recurrence in order to guide adjuvant therapy.

Lung tumor messenger ribonucleic acid (mRNA) profiling has been extensively investigated to identify single-genes or multi-gene signatures that provide prognostic information [3]. The rationale is that differentially expressed genes in lung cancers mirror different biological properties of the tumors, and thus, different prognoses observed in patients. So far, many studies were successful in using tumor-based gene expression biomarkers to improve risk-stratification after surgical resection. However, no gene or signature has been widely implemented in lung cancer clinical setting. This reflects the inherent challenges of identifying robust biomarkers that are transferable in real-life clinical setting. Validation in independent datasets is of paramount importance to select the best biomarkers for prospective clinical studies and ensure clinical translation. In this study, we attempted to validate genes with demonstrated prognostic value in two independent sets of patients with early-stage lung adenocarcinoma. We intentionally focused on single-gene biomarkers as feasibility and cost of practical assay development are more favorable than multi-gene signatures.

## Materials and methods

### Study participants

Lung tumor tissue was obtained from white French Canadian patients of European descent undergoing primary lung cancer surgery between 1999 and 2014 at the *Institut universitaire de cardiologie et de pneumologie de Québec* (IUCPQ). Fresh-frozen tumor specimens were taken from the IUCPQ site of the Respiratory Health Network Biobank of the *Fonds de la recherche en santé du Québec–Santé* (www.tissuebank.ca). Lung tumors for the first validation set were from 233 patients that underwent lung resection for pathologically confirmed stage I adenocarcinoma. Similarly, the second validation set consisted of 210 patients with stage I adenocarcinoma. The two validation sets were collected using the same conditions and procedures. However, the distribution in the years of patients’ surgery is later in the validation set 2, reflecting the periods we built these cohorts. Corresponding clinical variables including demographics, pathology report and smoking status were obtained from the biobank database. Patients’ medical charts were abstracted for follow-up starting at the time of surgery, vital status, date and cause of death. Patients were observed until death or last follow-up. Exclusion criteria for the two validation sets include never smokers, positive neoplastic margins on resected lung tissue and previous cancer of any origins with systemic treatment within 5 years of lung cancer surgery. Never smokers were excluded as lung cancer development in these individuals is considered a distinct entity [4, 5] with different tumor-based gene expression profile [6] and survival [7]. Staging was performed using the 6^th^ edition of the TNM Classification of Malignant Tumours [8] for samples obtained between 1999 and 2009, and the 7th edition [9] for samples obtained in 2010 and after. By reclassifying all patients to the 7^th^ edition, 16 patients in validation set 1 and two patients in validation set 2, originally considered stage I based on the 6^th^ edition, were reclassified as stage II owing to a tumour size greater than five centimeters. Sensitivity analysis excluding these patients indicates no significant difference in prognostic values for *EZH2* and *RRM1*. Lung tissue samples were obtained in accordance with Institutional Review Board guidelines. All patients provided written informed consent, and the ethics committee of the *Institut universitaire de cardiologie et de pneumologie de Québec* approved the study (#20968). The clinical characteristics of patients in the two validation sets are shown in **Table 1**.

**Table 1:** Clinical characteristics of the patients that underwent curative intent resection for stage I adenocarcinoma.

	Validation set 1 (n = 233)	Validation set 2 (n = 210)	All (n = 443)
**Age (years)**	63.8 ± 9.0	64.3 ± 8.6	64.1 ± 8.8
**Sex**			
Male	112 (48.1%)	92 (43.6%)	204 (46%)
Female	121 (51.9%)	118 (56.4%)	239 (54%)
**Smoking status**			
Current-smoker	72 (30.9%)	51 (24.2%)	123 (27.8%)
Ex-smoker	161 (69.1%)	159 (75.8%)	320 (72.2%)
**Histology**			
Adenocarcinoma	233 (100%)	210 (100%)	443 (100%)
**Stage**			
IA	104 (44.6%)	109 (51.9%)	213 (48.1%)
IB	129 (55.4%)	101 (48.1%)	230 (51.9%)
**Tumor size (cm)**			
≤ 3	144 (61.8%)	142 (67.6%)	286 (64.6%)
>3 - ≤5	71 (30.5%)	65 (30.9%)	136 (30.7%)
>5 - ≤7	11 (4.7%)	2 (1.0%)	13 (2.9%)
>7	5 (2.1%)	1 (0.5%)	6 (1.4%)
Unknown	2 (0.9%)	0 (0%)	2 (0.4%)
**Follow-up censored at 5 years (months)**	53.7 ± 14.9	47.8 ± 14.3	49.9 ± 14.7
**Deaths at 5 years (n)**	62 (27%)	33 (15%)	95 (21.4%)

Continuous variables are mean ± standard deviation (SD)

### Lung tumor specimens

Within 30 minutes following surgical resection, a pulmonary pathologist immediately examined lung specimens for diagnosis purposes, and collected tumor tissue for the biobank. The research specimens were immediately divided into smaller fragments (~0.5cm^3^) placed in 5mL cryovials and snap-frozen in liquid nitrogen. The cryovials were then transported in dry ice to the IUCPQ Biobank where they were stored at -80°C until further processing. A representative histologic slide of tumor tissue was reviewed by a pathologist (P.J.) to ensure high tumor cell content.

### Candidate prognostic genes

**Fig 1:**
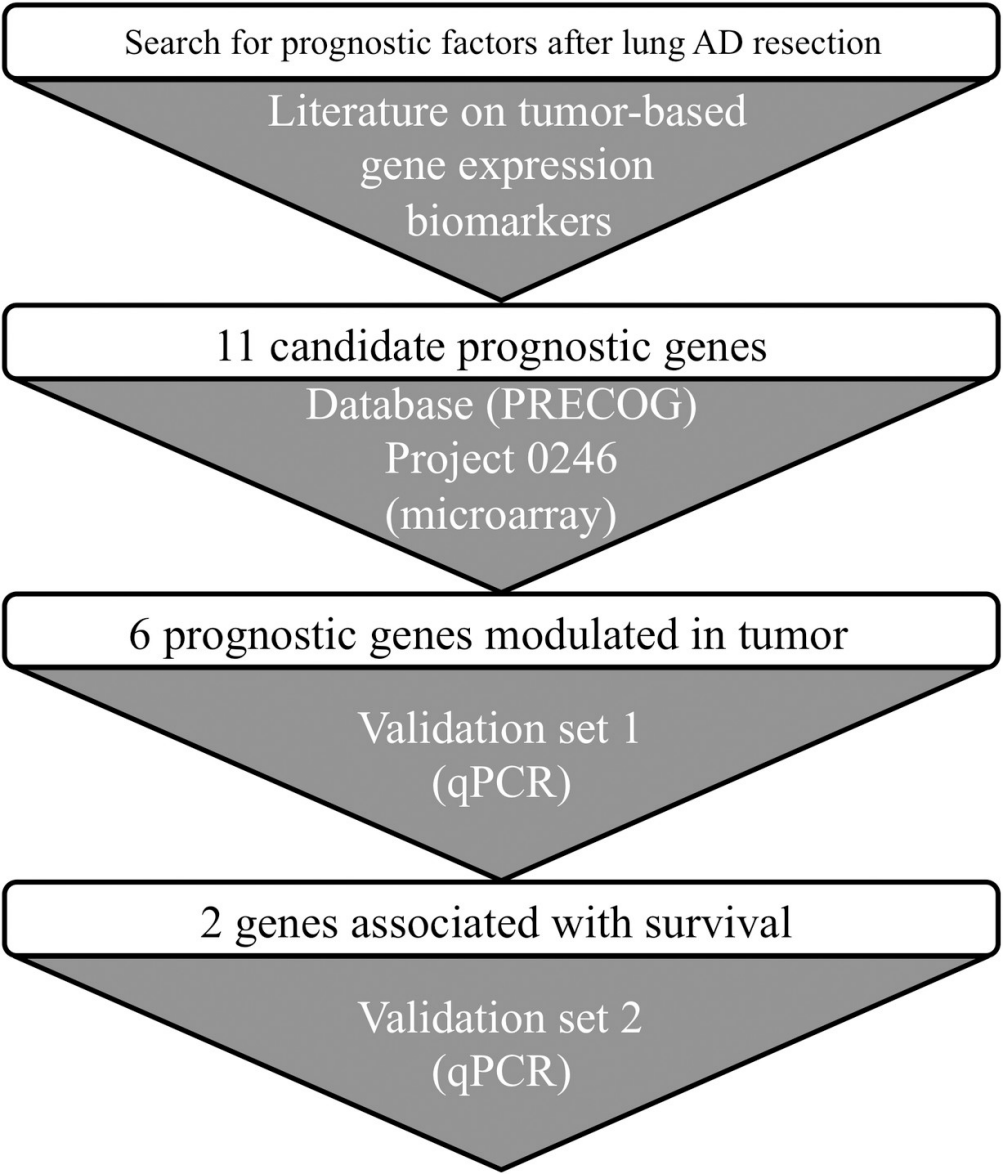
Flowchart for gene expression biomarkers selection, filters, and analysis.

An overview of the strategy to select candidate prognostic genes is illustrated in **Fig 1**. Selection was restricted to mRNA expression levels of single-genes measured in lung tumor that had demonstrated association with survival. Eleven candidate genes were selected from the literature (**Table 2**). PREdiction of Clinical Outcome from Genomics profiles (PRECOG) was then used to filter the gene selection. PRECOG is a pan-cancer resource to evaluate prognostic value of gene expression from publically available datasets (https://precog.standford.edu) [10]. Prognostic significance in PRECOG is evaluated using meta-z-scores, which consist of meta-analysis of z-scores derived from individual studies for each gene in each cancer type. In this study, meta-z-scores specific for lung adenocarcinoma were queried and candidate genes with meta-z-score of poor (meta-z-scores>3.0) and good (meta-z-scores<-3.0) survival were retained. Genes passing this filter were then evaluated in our previous microarray-based study comparing gene expression of resected lung adenocarcinoma to adjacent non-tumor pulmonary parenchyma collected at 0, 2, 4 and 6 cm from the lesion in 12 patients [11]. In this dataset (available in GEO: GSE83213), we specifically assessed whether prognostic candidate genes associated with poor and good survival were concordantly up- and down-regulated in tumor compared to non-tumor lung tissue, respectively.

**Table 2:** Investigation of selected prognostic tumor-based gene expression biomarkers in PRECOG.

Symbol	Name	Reference	PRECOG
***FOXM1***	Forkhead box M1	Kong et al. Oncology Reports 2014 [13]	**7.2**
***EZH2***	Enhancer of Zeste 2 polycomb repressive complex 2 subunit	Behrens et al. Clin Cancer Res 2013 [14]	**5.2**
***RRM1***	Ribonucleotide Reductase catalytic subunit M1	Bepler et al. J Clin Oncol 2004 [15]	**5.0**
***PPARG***	Peroxisome Proliferator-Activated Receptor gamma	Sasaki et al. Lung Cancer 2002 [16]	**2.7**
***HOXB2***	Homeobox B2	Inamura et al. J Thorac Oncol 2007 [17]	**0.4**
***ERCC1***	Excision Repair Cross-Complementation group 1	Simon et al. CHEST 2005 [18]	**-0.1**
***TIMP3***	Tissus Inhibitor of Metalloproteinas-3	Mino et al. J Surg Oncol 2007 [19]	**-0.4**
***EIF3E***	Eukaryotic translation Initiation Factor 3 subunit E	Buttitta et al. Clin Cancer Res 2005 [20]	**-1.1**
***NFIB***	Nuclear Factor I/B	Becker-Santos et al. J Pathol 2016 [21]	**-3.2**
***SELENBP1***	Selenium-Binding Protein I	Chen et al. J Pathol 2004 [22]	**-6.1**
***BTG2***	B-cell Translocation Gene 2	Wei et al. Tumor Biol 2012 [23]	**-6.5**

### Gene expression measurements

RNA was extracted from 30 mg of frozen lung tissue using the RNeasy Universal Plus Mini kit (Qiagen). RNA concentration and purity were assessed by UV 260 nm and UV 260/280 nm ratio respectively with the NanoVue spectrophotometer (GE Healthcare). Two micrograms of RNA were converted to cDNA using QuantiTect Reverse Transcription kit (Qiagen). qPCR was performed using the SsoAdvanced Universal SYBR Green Supermix (Bio Rad) on the Bio Rad CFX384 Real-time PCR system for *BTG2*, *SELENBP1*, *NFIB*, *RRM1* and *EZH2*. For *FOXM1*, the PowerUp SYBR Green Master Mix (Thermo Fisher Scientific) was used instead. The reaction volume was 10μL. Cycling steps were 1 cycle of 30 sec at 95°C then 39 cycles of 15 sec at 95°C and 30 sec at 60°C. *SELENBP1* and *FOXM1* were amplified using a touchdown cycling program (**S1 Table**). Four reference genes were considered including *GAPDH*, *ACTB*, *BAT1* and *B2M*. The primers were designed manually and synthesized by Integrated DNA Technologies (Toronto, Ontario). PCR primers were tested *in silico* using BLAT in UCSC (www.genome.ucsc.edu/cgi-bin/hgBlat) to confirm their binding to a unique region of the human genome and the absence of underlying polymorphisms. Primers for target and reference genes, amplicon sizes, and annealing temperatures are shown in **S1 Table**. For each gene, amplification conditions (annealing temperature, specificity, efficiency) were validated according to the MIQE guidelines [12]. For each gene, the experimental samples were tested in triplicate. The cDNA copy number of each sample were calculated according to the standard curve method and normalized to the average copy number of the four reference genes.

### Statistical analyses

Our primary endpoint was overall survival (OS) from the time of surgery to death from any cause. The follow-up was censored at five years. Disease-free survival (DFS) was a secondary endpoint and defined as the time of surgery to recurrence. Univariate cox proportional hazards regression analyses were performed to assess the association between gene expression and survival. Median of normalized gene expression was used as cutoff to separate patients with high and low gene expression. Survival probabilities were estimated using Kaplan-Meier analysis and log-rank test was used to assess the difference between survival curves. Multivariate Cox proportional hazards regression models were conducted to assess whether genes associated with survival were independent of clinical parameters including pathological stage, age, and sex. Relationships between mRNA expression levels of genes were evaluated by Spearman correlation. All statistical tests were two-sided and p-values < 0.05 were considered significant. All analyses were carried out with R statistical software version 3.2.2. Kaplan-Meier analysis and Cox proportional hazards regression models were performed using the R package *survival*.

## Results

### Candidate prognostic genes

**Fig 2:**
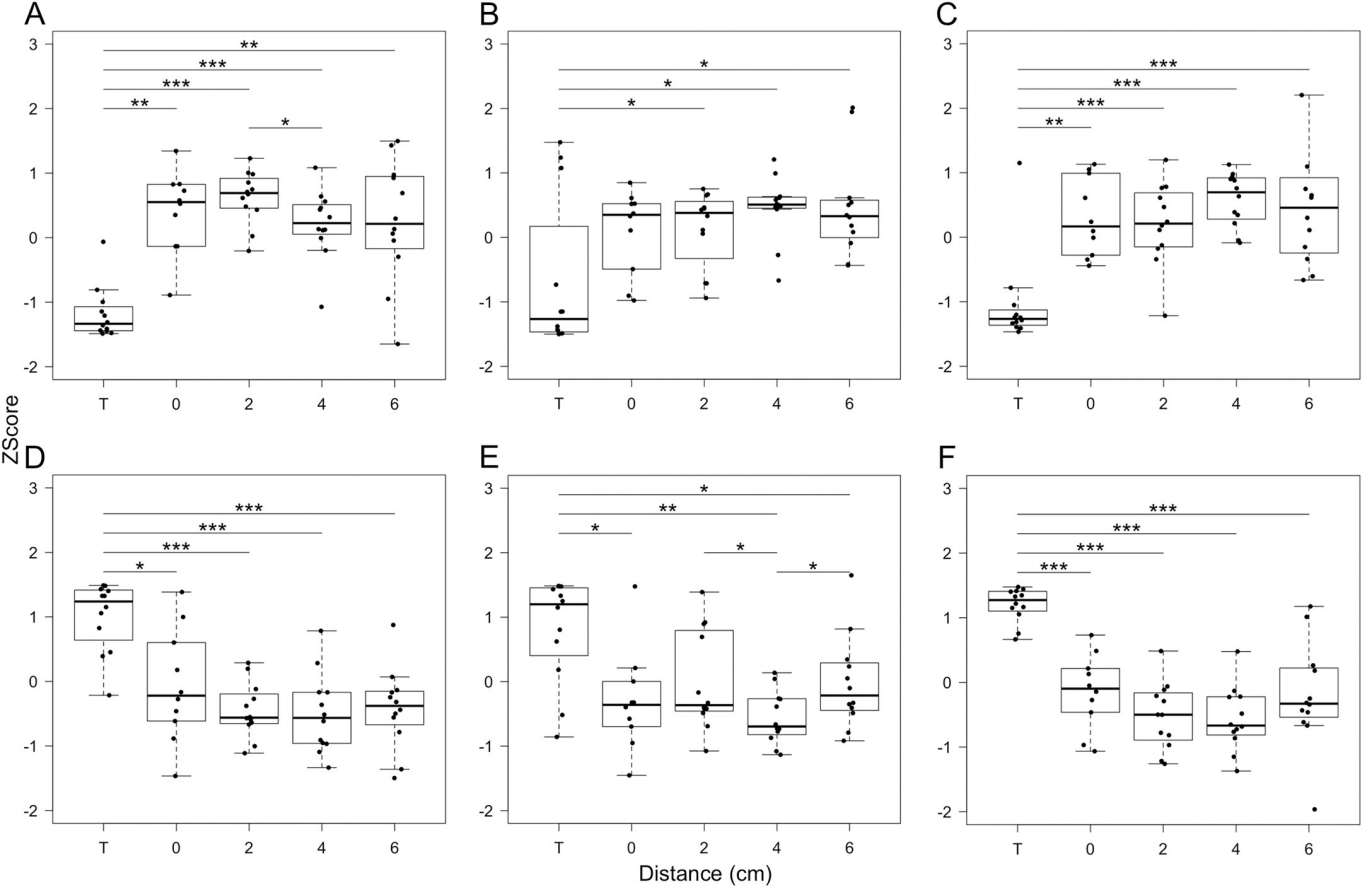
**Boxplots of gene expression levels of selected prognostic genes including *NFIB* (A), *SELENBP1* (B), *BTG2* (C), *FOXM1* (D), *RRM1* (E), and *EZH2* (F) in tumor and adjacent non-tumor lung tissue at different distances from the tumor.** The y-axis represents mean standardized gene expression values. The x-axis represents the geographical tissue samples at the tumor (T) and non-tumor sites at 0, 2, 4, and 6 cm away from the tumor (n = 12). Box boundaries, whiskers and centre mark in boxplots represent the first and third quartiles, the most extreme data point which is no more than 1.5 times the interquartile range (IQR), and median, respectively. *p<0.05, **p<0.01, ***p<0.001.

Eleven genes with demonstrated prognostic value based on mRNA expression levels in lung tumor were selected from the literature [13–23] (**Table 2**). The prognostic value of six of these genes was confirmed in PRECOG including three genes with good prognosis (meta-z-scores <-3.0: *BTG2*, *NFIB* and *SELENBP1*) and three genes with poor prognosis (meta-z-scores >3.0: *EZH2*, *RRM1* and *FOXM1*). Using our previous microarray dataset [11], we determined that the expression of *EZH2*, *RRM1* and *FOXM1* was significantly increased in tumor compared with the non-tumor lung tissue and the expression of *BTG2*, *NFIB* and *SELENBP1* was significantly decreased in the tumor compared to non-tumor (**Fig 2**).

### Association between gene expression and survival in validation set 1

The clinical characteristics of the 233 patients in the first validation set are indicated in **Table 1**. The median duration of follow-up was 5.6 years. None of the 233 patients received adjuvant chemotherapy and/or radiation therapy. Pathological stages (stage IA and IB) were significantly associated with survival (Kaplan-Meier log-rank p = 6.5e-3, HR = 2.08, 95% CI = 1.21–3.57). Univariate Cox proportional hazards model for overall survival were performed with continuous value of gene expression. There was no significant association between gene expression and survival for *FOXM1*, *SELENBP1*, *BTG2* and *NFIB* (**S1 Fig**). However, mRNA expression levels of *RRM1* and *EZH2* were significantly associated with survival (**Table 3**).

**Table 3:** Univariate and multivariate Cox regression analyses of prognostic genes for overall survival in validation set 1.

		Univariate analyses		Multivariate analyses								
				*EZH2*			*RRM1*			*EZH2* + *RRM1*		
Features	Categories	HR* (95% CI)	P value	HR* (95% CI)	P value	Overall P	HR* (95% CI)	P value	Overall P	HR* (95% CI)	P value	Overall P
**Overall survival**						1.15e-06			1.55e-05			1.21e-06
Age	< 65	2.17 (1.28–3.70)	0.0034	2.21 (1.29–3.80)	0.0041		1.95 (1.14–3.34)	0.015		2.17 (1.26–3.74)	0.0051	
Sex	Male	1.50 (0.91–2.48)	0.11	1.16 (0.70–1.94)	0.57		1.37 (0.82–2.30)	0.23		1.23 (0.73–2.07)	0.44	
Pathologic stage	IB	2.08 (1.21–3.57)	0.0066	1.91 (1.11–3.29)	0.02		1.94 (1.12–3.34)	0.017		1.91 (1.11–3.29)	0.019	
*EZH2*	Continuous variable	1.33 (1.16–1.52)	3.06e-05	1.35 (1.17–1.55)	3.0e-05					1.27 (1.07–1.51)	0.007	
*RRM1*	Continuous variable	1.22 (1.09–1.36)	3.1e-04				1.22 (1.09–1.37)	0.0006		1.091 (0.95–1.26)	0.23	

*HR = hazard ratio, CI = confidence interval

**Fig 3:**
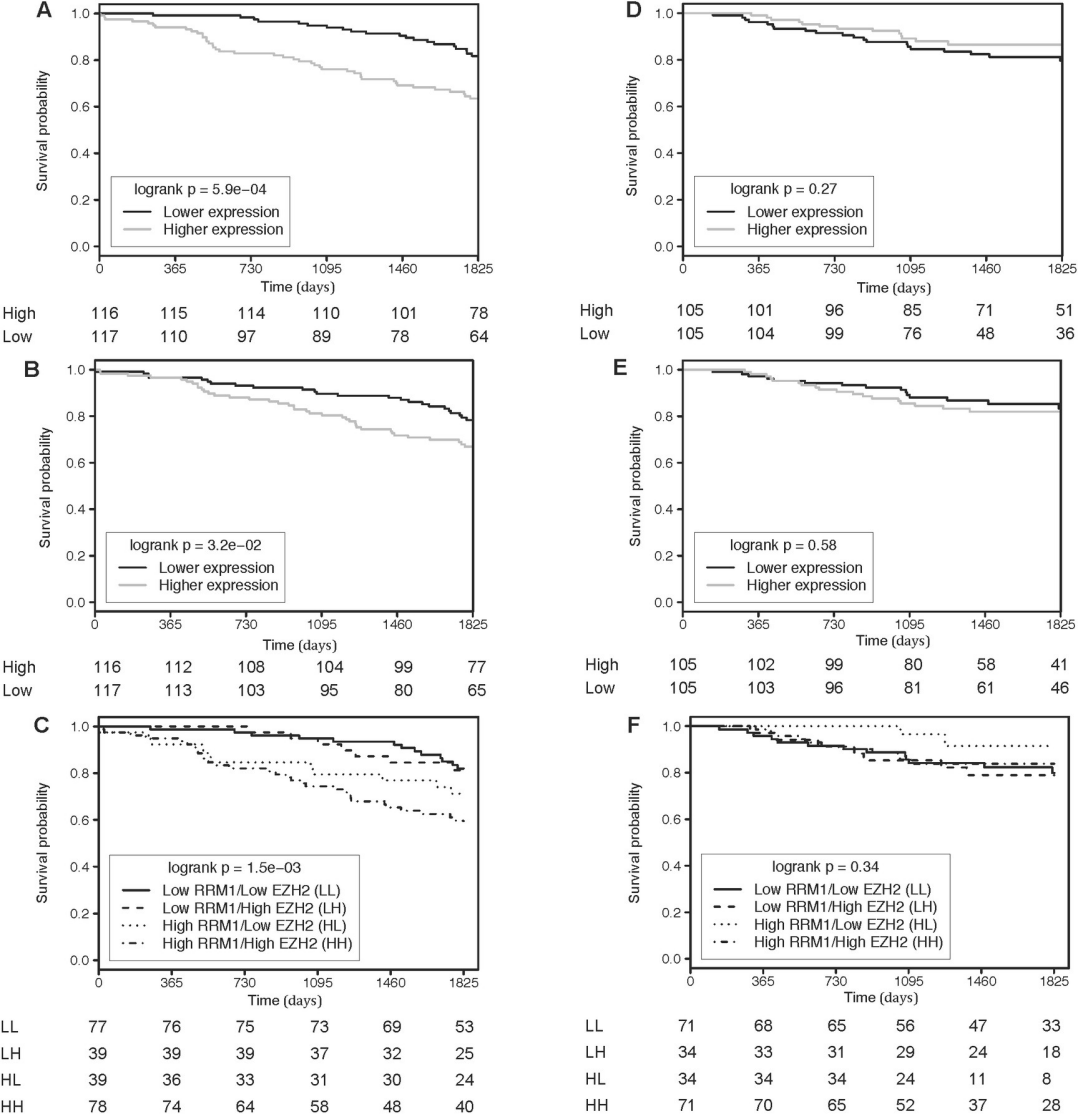
Kaplan-Meier analysis of overall survival in the validation set 1 according to median-derived risk categories for *RRM1* (A) and *EZH2* (B) and combination of *RRM1* and *EZH2* (C) and in the validation set 2 for *RRM1* (D), *EZH2* (E) and combination of *RRM1* and *EZH2* (F).

Patients were dichotomized into two groups based on the median value of gene expression of *RRM1* and *EZH2*. In univariate analysis, patients with high expression levels of *RRM1* and *EZH2* had significantly lower OS (*RRM1* Kaplan-Meier log-rank p = 5.9e-04, HR = 2.47, 95% CI = 1.45–4.20; *EZH2* Kaplan-Meier log-rank p = 3.2e-02, HR = 1.74, 95% CI = 1.04–2.90) (**Fig 3A** and **3B**) and lower DFS (*RRM1* Kaplan-Meier log-rank p = 9.1e-02, HR = 1.60, 95% CI = 0.92–2.78; *EZH2* Kaplan-Meier log-rank p = 4.1e-03, HR = 2.25, 95% CI = 1.27–3.98). In the validation set 1, 2- and 5-year survival rates of 88% and 67% were observed for patients with *EZH2* expression above the median compared to 93% and 79% for patients with *EZH2* expression below the median. For *RRM1*, survival rates at 2- and 5-years were 83% and 64% above the median and 98% and 83% below the median. Association with survival for *EZH2* and *RRM1* were confirmed in a multivariate analysis, after adjusting for age, sex and pathological stage (**Table 3**). Independent prognostic factors associated with overall survival in these models were age, pathologic stage and gene expression of *EZH2* or *RRMI*. Hazard ratios with 95% CI for risk groups defined by *EZH2* and *RRM1* expression levels in categories of age, sex, and stage are illustrated in **S2 Fig**. *RRM1* was associated with survival in both age groups (< or > 65 years old), in males and pathological stage IB. *EZH1* was associated with survival in younger patients and males.

There was a significant correlation between *RRM1* and *EZH2* expression in tumors of 233 patients (r = 0.47, p-value = 3.41e-14). We defined four groups of patients according to the combined expression of *RRM1* and *EZH2* using the same cutoffs based on the median of each gene. The frequency of patients in each group is the following: *RRM1*^Low^/*EZH2*^High^, N = 39, 17%; *RRM1*^Low^/*EZH2*^Low^, N = 77, 33%; *RRM1*^High^/*EZH2*^High^, N = 78, 33%; and *RRM1*^High^/*EZH2*^Low^, N = 39, 17%. Patients with tumor expressing *RRM1*^high^*/EZH2*^high^ had significantly worse OS (**Fig 3C**) and DFS rates than the 3 other groups (Kaplan-Meier log-rank p = 1.5e-03). At five years, the probability of survival was 60% in the *RRM1*^high^*/EZH2*^high^ group compared to 71–83% in the other groups.

### Association between gene expression and survival in validation set 2

The clinical characteristics of the 210 patients in the validation set 2 are indicated in **Table 1**. The median duration of follow-up was 4.7 years. None of the 210 patients received adjuvant chemotherapy and/or radiation therapy. The validation set 2 was similar to the validation set 1 regarding tumor histology, pathologic stage, sex, age and smoking-status (**Table 1**). However, the number of deaths at five years was lower in validation set 2 (15% compared to 27%). Survival curves between the two validation sets were different (S3 Fig), suggesting that patients from validation set 2 had a better outcome than patients from validation set 1. In contrast to validation set 1, pathological stages (stage IA and IB) were not significantly associated with survival in validation set 2 (Kaplan-Meier log-rank p = 0.41, HR = 0.75, 95% CI = 0.37–1.49).

Despite the limited number of events in validation set 2, we have attempted to replicate *RRM1* and *EZH2* using median-derived risk categories as performed above. In univariate analysis, there was no significant association between gene expression and survival for *EZH2* and *RRM1* (**Fig 3D** and **3E**). DFS was also similar between patients with high compared to low expression levels of these two genes (*RRM1* Kaplan-Meier log-rank p = 0.67, HR = 0.88, 95% CI = 0.50–1.56; *EZH2* Kaplan-Meier log-rank p = 1, HR = 1.00, 95% CI = 0.57–1.76). The lack of association with survival for *RRM1* and *EZH2* was also confirmed in multivariate analysis (not shown). Analyses by categories of age, sex, and pathological stage in validation set 2 did not corroborate observations made in validation set 1 (**S4 Fig**).

Again we observed a significant correlation between *RRM1* and *EZH2* expression in tumors from 210 patients (r = 0.58, p-value = 2.2e-16). We defined four groups of patients according to the combined expression of *RRM1* and *EZH2* as performed for validation set 1. The frequency of patients in each group is the following: *RRM1*^Low^/*EZH2*^High^, N = 34, 16%; *RRM1*^Low^/*EZH2*^Low^, N = 71, 34%; *RRM1*^High^/*EZH2*^High^, N = 71, 34%; and *RRM1*^High^/*EZH2*^Low^, N = 34, 16%. There was no significant association between the four groups and survival (**Fig 3F**).

### Joint analyses combining the two validation sets

**Fig 4:**
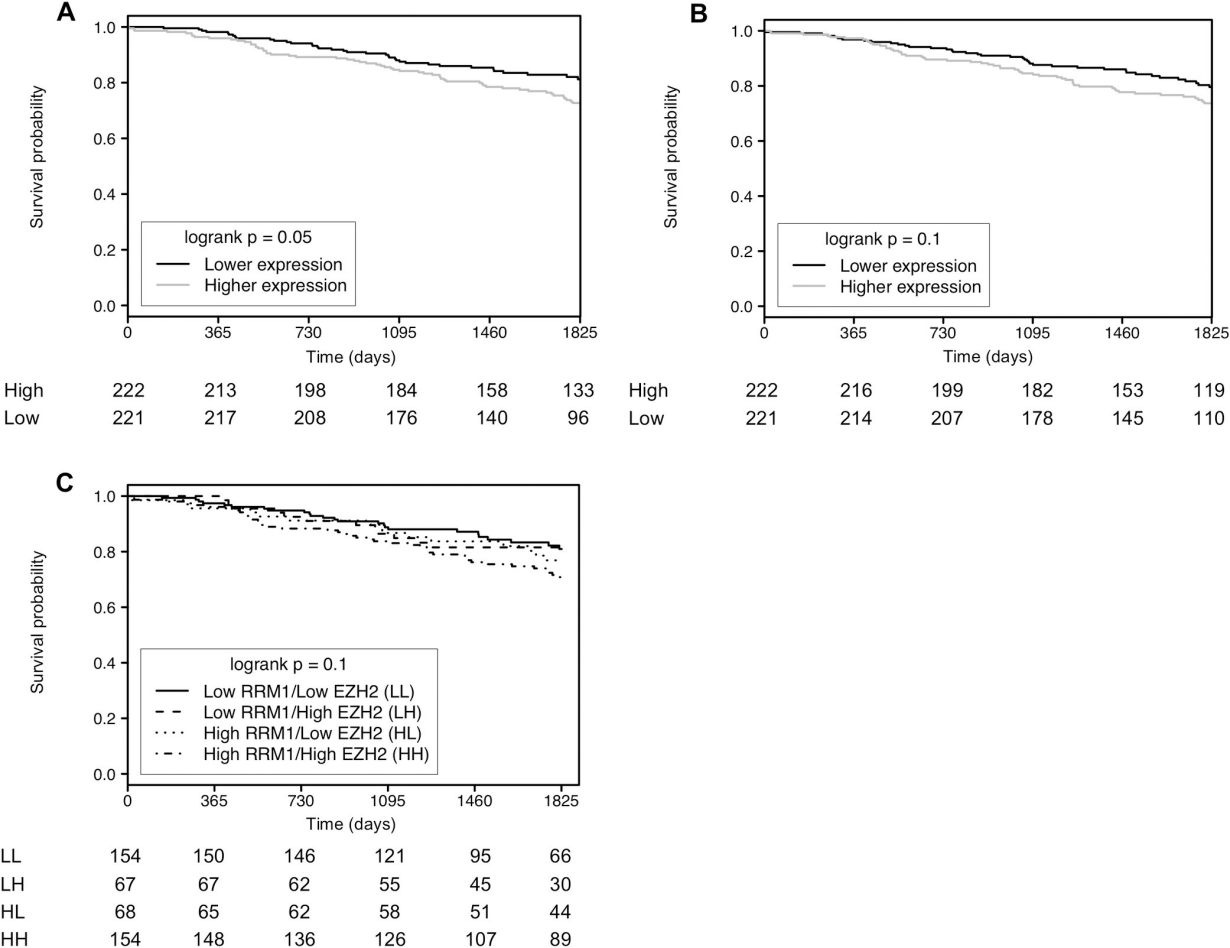
Kaplan-Meier analysis of overall survival in the combined set according to median-derived risk categories for *RRM1* (A), *EZH2* (B) and combination of *RRM1* and *EZH2* (C).

The clinical characteristics of the 443 patients in the combined validation sets are indicated in **Table 1**. The median duration of follow-up was 5.1 years. As performed above, patients in the combined set were dichotomized into two groups based on the median value of gene expression for *RRM1* and *EZH2*. For both genes, high expression levels were associated with worse survival, but the results did not reach statistical significance (**Fig 4A** and **4B**). As observed in both validation sets, there was significant correlation between *RRM1* and *EZH2* expression in tumors from 443 patients (r = 0.52, p-value = 2.2e-16). However, the four groups analysis based on the median levels of *RRM1* and *EZH2* were not associated with survival (**Fig 4C**).

## Discussion

Oncological outcome varies within 5 years after potentially curative surgical treatment in stage I lung adenocarcinoma, even in patients with similar clinical and pathological characteristics. This study, investigated the prognostic value of mRNA expression levels of candidate genes in tumor from patients treated by surgical resection. Genes with demonstrated prognostic value were investigated in two independent validation sets. Two genes, namely *RRM1* and *EZH2*, were associated with survival in the first validation set and were shown to provide prognostic value beyond standard clinical and pathological information. Higher expression of both genes was associated with decreased overall and disease-free survival. An attempt was made to replicate the results in a second validation series of stage I lung adenocarcinoma, but neither *RRMI* nor *EZH2* were associated with survival in this second set.

There is an urgent need to develop prognostic differentiation of patients with early-stage lung cancer beyond conventional clinicopathological TNM staging in order to guide complementary therapy during follow-up. This clinical need has led to the discovery of many biomarkers and prognostic classifiers of low- and high-risk of postoperative mortality. Unfortunately, none have been adopted in clinical setting. To become standard of practice, these biomarkers/classifiers must be validated and developed into an available product to treating physicians. The primary objective of this study is to provide further validation of known single-gene prognostic biomarkers in order to advance towards clinical translation and clinical implementation. However, our study has demonstrated that tumor-based gene expression biomarkers are challenging to develop and validate.

We have obtained encouraging results for *EZH2* in the first validation set. Our results are consistent with previous studies showing worse outcomes in patients with high levels of *EZH2* protein expression [14]. In validation set 1, we showed that *EZH2* mRNA expression was able to predict patient survival on top of clinical variables, but this was not confirmed in validation set 2. A trend was observed in the joint analyses (n = 443) suggesting that a larger sample size may be required to demonstrate the prognostic value of *EZH2*. It should be noted that *EZH2* has recently been considered as part of a five protein expression classifiers that has failed to outperform clinical parameters [24].

The expression of *RRM1* was also associated with survival in validation set 1. However, the direction of effect was not consistent with the study by Bepler and colleagues showing *RRM1* expression as a predictor of good outcome for patients with lung cancer [15]. In the later study, different histology subtypes of non-small-cell lung cancer and pathological stages were considered as well as patients with mixed smoking history, i.e. never, former and current smokers. Gene expression patterns differ between histology subtypes of lung cancer [25, 26] and smoking status [6, 27]. Accordingly, selection of patients may, at least in part, explain the different results. In our study, we have focused specifically on stage I adenocarcinoma in ever-smokers with the hope to homogenize the population and facilitate validation. Although our two validation sets were clinically and pathologically similar, the prognostic value of *RRM1* observed in validation set 1 was not replicated in validation set 2. Again, the joint analysis showed a trend that may worth pursuing in a larger cohort.

This study has limitations. A lower number of events occurred in validation set 2, which limited our power to identify any prognostic factors. Analyses performed in validation set 2 will need to be repeated with a longer follow-up. qPCR was performed in fresh frozen tissues, which are not available in most community-based hospitals. Validation of tumor-based gene expression biomarkers in formalin-fixed and paraffin-embedded tissues (FFPE) would facilitate widespread applicability. This step was part of our developmental pipeline, but for biomarkers that demonstrated sufficient value in fresh frozen tissues. Here, we intentionally focused on single-gene biomarkers to facilitate practical assay development. Although the reproducibility and clinical utility of multi-gene signatures have been questioned [28], such signatures reflecting diverse biological processes may prove to have more predictive value. Finally, we have considered only one omic dimension, i.e. gene expression. Multi-omics molecular information (e.g. somatic mutations and methylation) is likely to be more successful to develop valuable prognostic classifiers. The EGFR mutational status was not available in our study. Although still controversial, some lines of evidence suggested better survival in EGFR positive patients [29, 30]. At our institution, EGFR mutational status is only tested in advanced-stage lung cancer with an adenocarcinoma component. Our two sets of patients are characterized by early-stage lung adenocarcinoma and received no pre- or postoperative therapy. Accordingly, we do not have the EGFR mutational status in our cohorts.

Our study further highlights challenges to develop prognostic classifiers capable of delineating recurrent and non-recurrent early-stage lung cancer. Here, tumor specimens for both validation sets were obtained using standardized methods at a single site. As aforementioned, the clinical and pathological characteristics of both sets were matched. Despite these efforts, we were unable to validate some of the most promising single-gene prognostic biomarkers. Molecular profiling in tumor samples will remain difficult owing to intratumor heterogeneity [31]. Accordingly, others have started to use other medias to derive robust prognostic classifiers [32, 33]. Future studies will need to evaluate diverse molecular phenotypes (e.g. gene expression, somatic mutations and methylation) but also diverse biospecimen medias including tumor, adjacent non-tumor lung specimens, and liquid biopsies. Comprehensive biological sample collection and large sample size will be required.

## Conclusion

*EZH2* and *RRM1* mRNA expression in resected stage I adenocarcinoma were associated with survival in a first validation set, but not replicated in a clinically and pathologically matched independent set. A trend was observed in the combined dataset for both genes, which calls for larger sample size of patients to identify prognostic biomarkers. This study further highlights challenges to identify prognostic biomarkers following early-stage lung cancer resection. Diverse molecular phenotypes and biospecimen medias will need to be considered to develop classifiers capable to improve postoperative risk-stratification and more accurately identify patients with early-stage pulmonary adenocarcinoma that may benefit from adjuvant therapy.

## Supporting information

S1 Fig Kaplan-Meier analysis of overall survival according to median-derived risk categories for *BTG2* (A), *SELENBP1* (B), *NFIB* (C), and *FOXM1* (D).
(TIFF)

S2 Fig Forest-plot of hazard ratios for overall survival in categories of age, sex and pathological stage in validation set 1. (TIFF)

S3 Fig Kaplan-Meier analysis comparing overall survival between the two validation sets. (TIFF)

S4 Fig Forest-plot of hazard ratios for overall survival in categories of age, sex and pathological stage in validation set 2. (TIFF)

S1 Table Primers, amplicon sizes, and annealing temperatures for target and reference genes. (TIFF)
