# Supplementary figures and images for: Tumor-based gene expression biomarkers to predict survival following curative intent resection for stage I lung adenocarcinoma
Source: PLoS One. 2018 Nov 20;13(11):e0207513. doi: 10.1371/journal.pone.0207513 (PMC6245750; doi:10.1371/journal.pone.0207513)

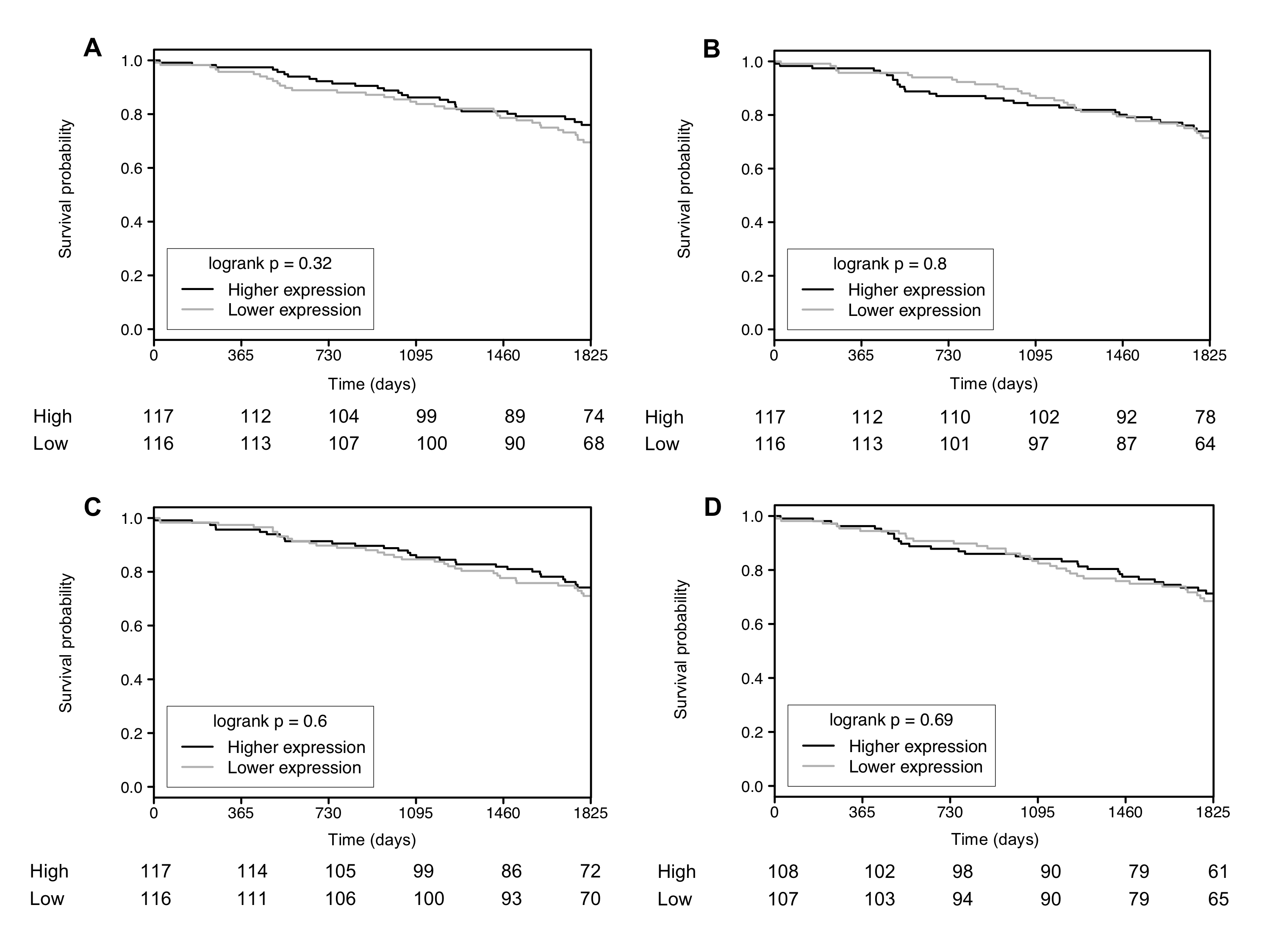

Supplement: S1 Fig — Kaplan-Meier analysis of overall survival according to median-derived risk categories for BTG2 (A), SELENBP1 (B), NFIB (C), and FOXM1 (D). (TIFF) [file pone.0207513.s001.tiff]

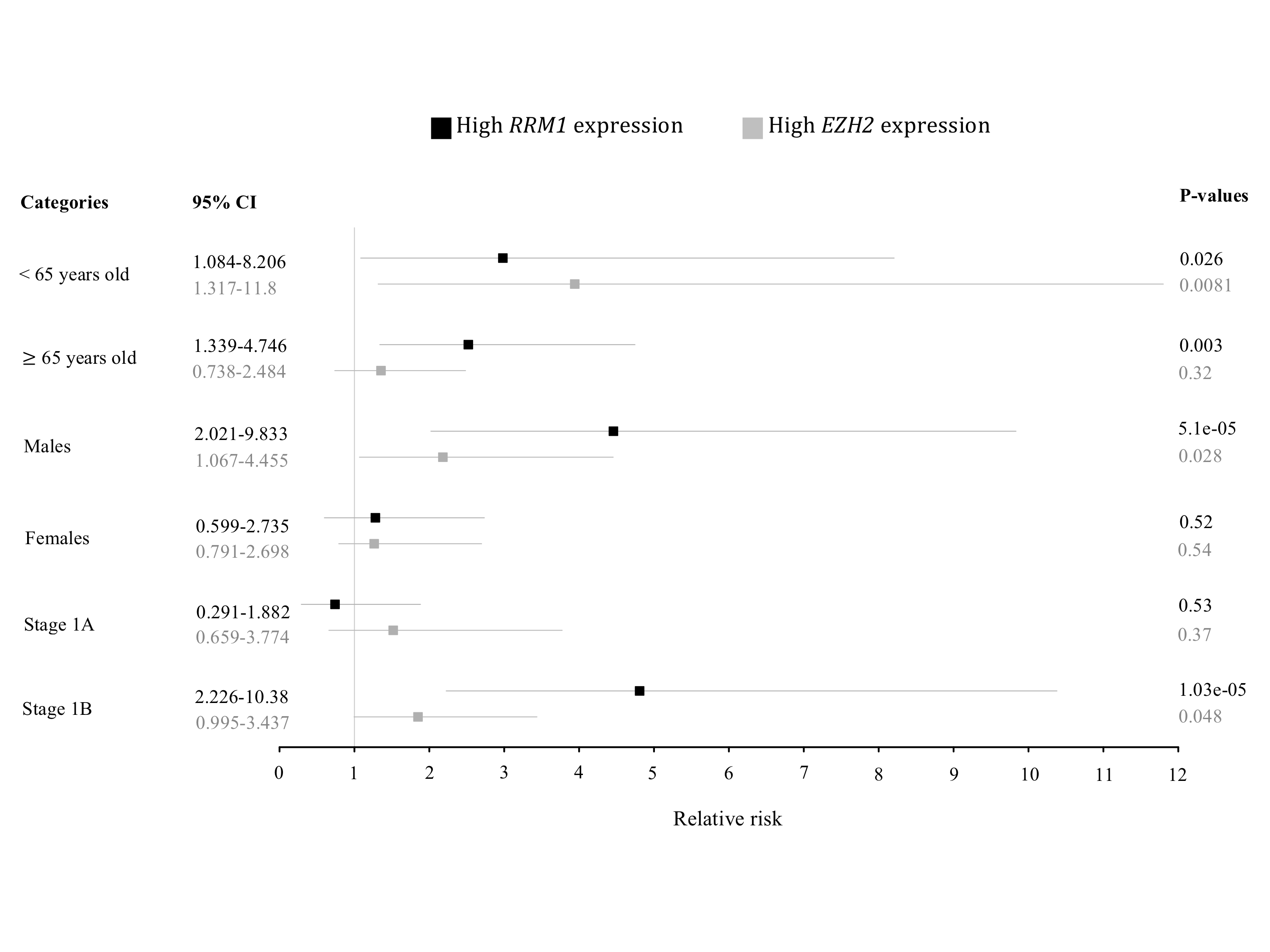

Supplement: S2 Fig — (TIFF) [file pone.0207513.s002.tiff]

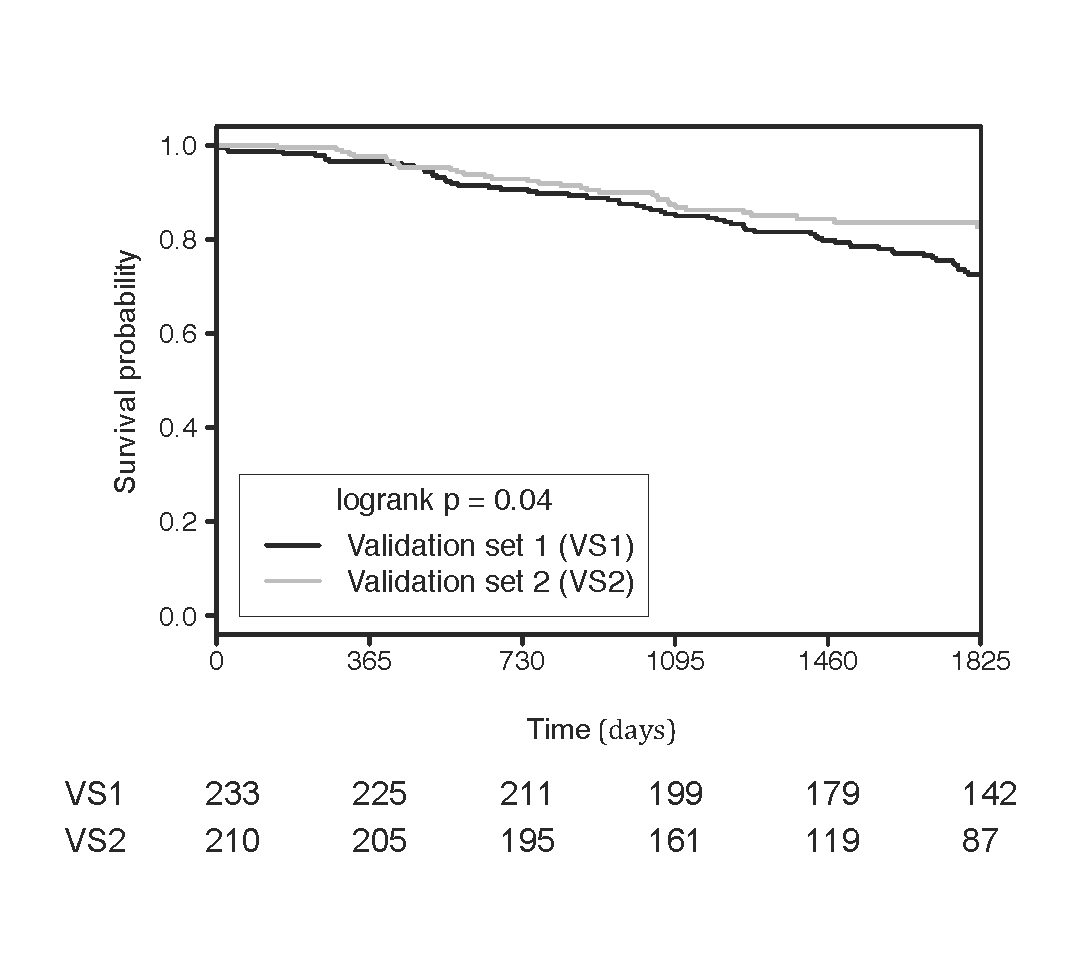

Supplement: S3 Fig — (TIFF) [file pone.0207513.s003.tiff]

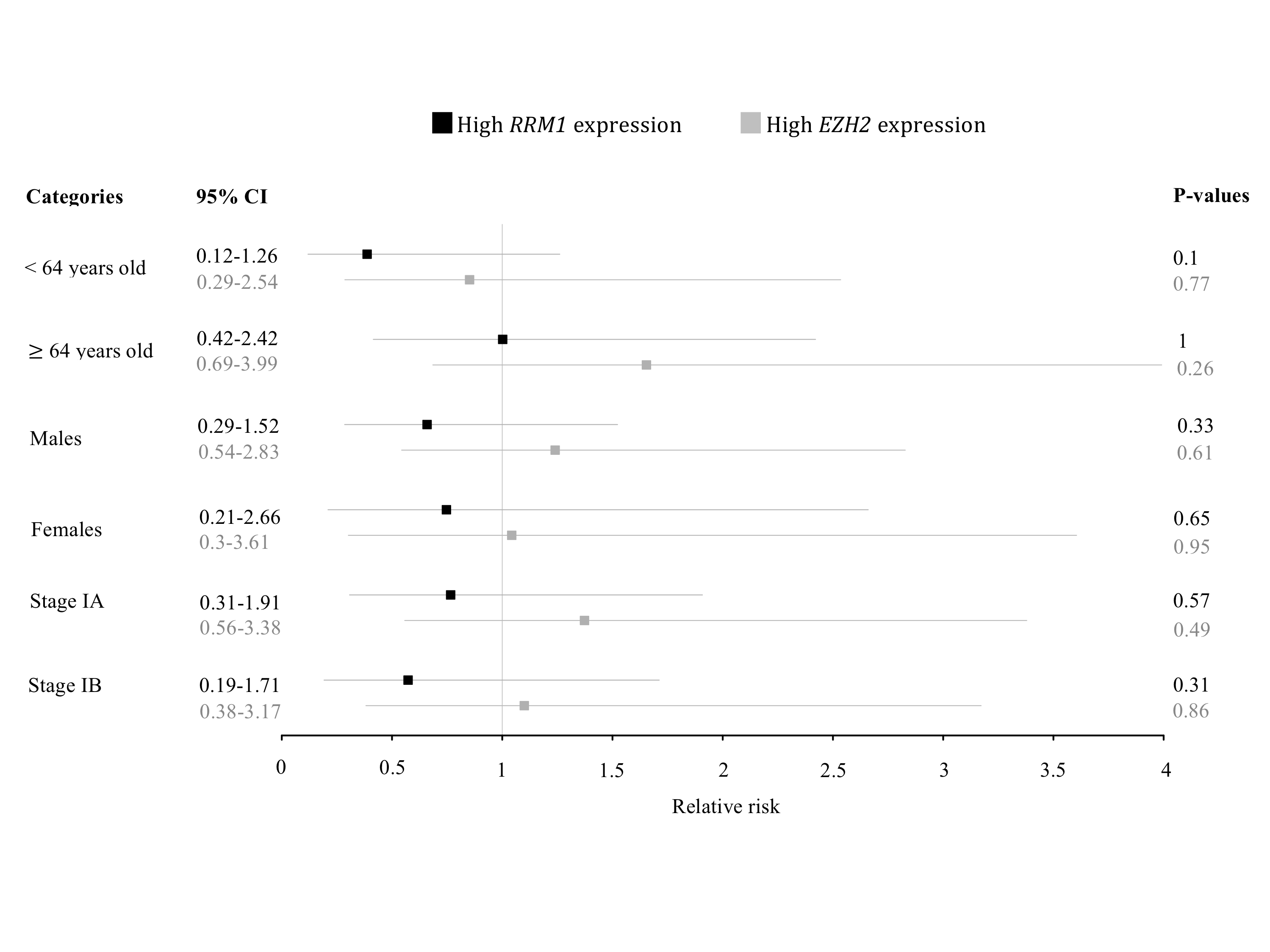

Supplement: S4 Fig — (TIFF) [file pone.0207513.s004.tiff]

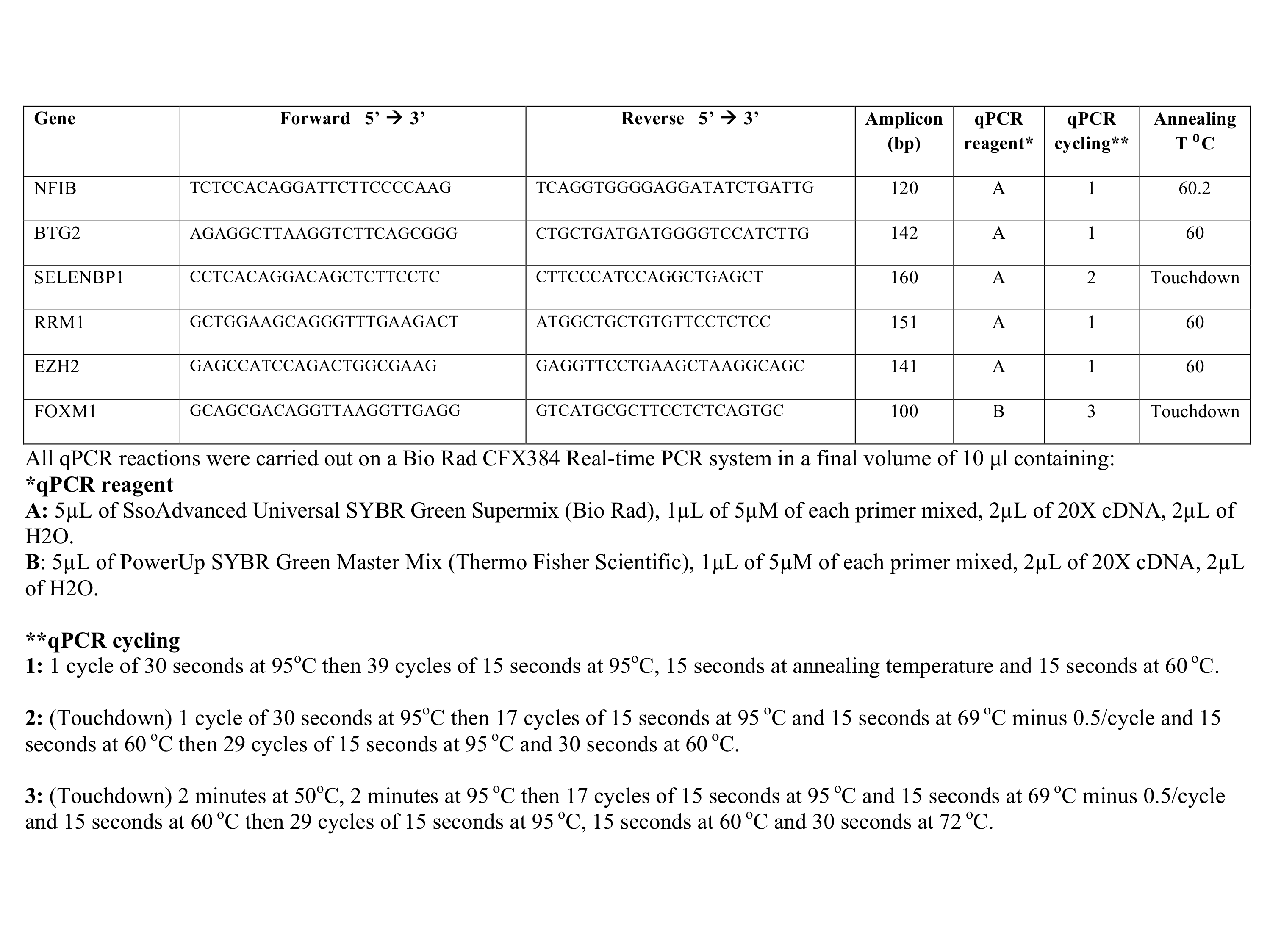

Supplement: S1 Table — (TIFF) [file pone.0207513.s005.tiff]
